# Chlorate of Pottassa, Etc.

**Published:** 1871-10

**Authors:** A. H. Brantley

**Affiliations:** Decatur, Ga.


### CHLORATE OF POTASSA. IS IT A PARTURIFACIENT?

By A. H. Brantley, M. D., Decatur, Ga.

In this age of human progress, while busy brains are hard at work, and toiling hands are never tired, exploring the wonderful capabilities of nature, we are prone to be thrown off our guard by a laudable enthusiasm over constantly recurring novelties.

The wisest and best of our profession, ever busy in the many broad fields of Medical Science, are gathering in every land, and on every sea, new facts, searching out new truths, applying new principles, evolving new laws, and with wonderful rapidity continually flashing new and startling discoveries over the medical world.

While every medical man who loves his noble calling must feel a just pride in these evidences of *real* progress, at least in Medical Science; yet the candid mind will see in this very advance, the necessity for a closer and more careful circumspection than ever before. Bewildered by the dazzling array of therapeutical agents that are crowding themselves upon our notice, too many of which bear within themselves the painful evidence of having sprung from that idle and impossible dream of a specific therapy, the young practitioner certainly runs the risk of losing sight of those common, well known remedies that have for years been in general use among physicians. The temptation to use and rely upon novelties, is perhaps greater, in the practice of medicine than in any other avocation in life.

The desire to warn the younger members of our profession from relying too much on new remedies to the exclusion of the old and well-tried ones has, perhaps, too long kept us from the main object of this paper, viz: to call the attention of the profession to some therapeutical properties of one or two medical agents that have not heretofore generally been thought to possess these properties. We would here state by way of contributing our mite towards giving efficiency to the thought that if the profession generally would, in the exhibition of our more common remedies, watch their therapeutical action with the same eagerness and care that they now do some of the newer agents on trial, there is little hazard in saying that many new and valuable discoveries would result.

We could probably give point to the question which heads this article, in no way more directly than by giving, of the number of cases in our possession, the history of one or two:

### Case I.

*June* 16*th*, 1868, was consulted by her husband, on the case of Mrs. Mary R——, age 22 years, a stout, healthy woman, weighing about 130 pounds, had been married two years, but had never, as her husband stated, been pregnant; her menses had appeared regularly after marriage, until two months before the date above given. Since then there had been, as she said "no show." Had been well after marriage up to disappearance of catamenia and was well even then, with the exception of about five days during the time this discharge should have appeared, at which time she suffered greatly with pain in the head, back and limbs, dizziness, tremors, nausea, etc. Declined doing anything until a thorough examination of the case could be made, which was had on the same day. A careful examination (taxis omitted) failed to give any indications of pregnancy. As she was nearly through the usual period of suffering, I prevailed on her to wait until the next month, promising that if the trouble returned again I would interfere. At the anticipated time the same trouble occurred again. I gave her bromide of potassium in five grain doses with the most prompt and decided relief. So great was the relief the lady asked me the name of the medicine with the proper proportions for combining, and the dose, which I gave her. In one week she sent to the neighboring village for the bromide, but her husband having left the prescription at home was compelled to trust to memory and bought chlorate potassium instead of the bromide. On the same evening she took one dose of the chlorate, probably seven, eight or ten grains at about 2 o'clock, P. M. At 3½ o'clock, P. M., I was summoned in haste. Saw her at 4 o'clock, P. M.: Uterus contracting energetically, with a fœtus at about two and a half months extruding from the os. Fœtus showed no arrest of development of any kind, nor was there in its appearance anything explanatory of the cause of the abortion. The patient said that she had felt no pain, sickness or inconvenience of any kind up to the moment of taking the medicine, but that "the bromide" had made her feel so very comfortable that she wished to take it again. She was intelligent and expressed herself as very greatly disappointed at the occurrence of the accident; as surprised at her condition as relieved by it, and very desirous of becoming a mother. She most positively declares that she had taken no violent exercise or otherwise injured herself in any way, so far as she was aware. I satisfied myself as to the genuineness of the chlorate potass., both by specimens of that purchased and by consulting the druggist from whom it was obtained. I believe the lady's statements.

The succeeding case (No. 2,) will be given in the next number of this Journal, together with some deductions and comments based upon the facts detailed.

